# Inhibition of Epithelial TNF-α Receptors by Purified Fruit Bromelain Ameliorates Intestinal Inflammation and Barrier Dysfunction in Colitis

**DOI:** 10.3389/fimmu.2017.01468

**Published:** 2017-11-10

**Authors:** Zijuan Zhou, Liang Wang, Panpan Feng, Lianhong Yin, Chen Wang, Shengxu Zhi, Jianyi Dong, Jingyu Wang, Yuan Lin, Dapeng Chen, Yongjian Xiong, Jinyong Peng

**Affiliations:** ^1^Laboratory Animal Center, Dalian Medical University, Dalian, China; ^2^College of pharmacy, Dalian Medical University, Dalian, China; ^3^Dalian Medical University, Dalian, China; ^4^Central Laboratory, the First Affiliated Hospital, Dalian Medical University, Dalian, China; ^5^College of Integrative Medicine, Dalian Medical University, Dalian, China

**Keywords:** bromelain, purification, inflammation, cytokines, myosin light chain kinase, TNF-α receptor, inflammatory bowel disease, tight junction

## Abstract

Activation of the TNF-α receptor (TNFR) leads to an inflammatory response, and anti-TNF therapy has been administered to reduce inflammation symptoms and heal mucosal ulcers in inflammatory bowel disease (IBD). Bromelain, a complex natural mixture of proteolytic enzymes, has been shown to exert anti-inflammatory effects. This study aimed to investigate the effect of purified fruit bromelain (PFB)-induced inhibition of epithelial TNFR in a rat colitis model. Colitis was established by intracolonic administration of 2, 4, 6-trinitrobenzene sulfonic acid. Expression of TNFR1 and TNFR2 was measured by quantitative RT-PCR and western blotting. The effect of PFB on colitis was evaluated by examining the inflammatory response and intestinal epithelial barrier function. Our results showed that both TNFR1 and TNFR2 expression were significantly increased in a colitis model, and the increase was significantly reversed by PFB. Colitis symptoms, including infiltration of inflammatory cells, cytokine profiles, epithelial cell apoptosis, and epithelial tight junction barrier dysfunction were significantly ameliorated by PFB. Compared with fruit bromelain and stem bromelain complex, the inhibition of TNFR2 induced by PFB was stronger than that exhibited on TNFR1. These results indicate that PFB showed a stronger selective inhibitory effect on TNFR2 than TNFR1. In other words, purification of fruit bromelain increases its selectivity on TNFR2 inhibition. High expression of epithelial TNFRs in colitis was significantly counteracted by PFB, and PFB-induced TNFR inhibition ameliorated colitis symptoms. These results supply novel insights into potential IBD treatment by PFB.

## Introduction

Inflammatory bowel diseases (IBDs) are a group of recurrent inflammatory conditions of the colon and small intestine mainly of ulcerative colitis and Crohn’s disease (1). IBDs are important diseases of the gastrointestinal tract and they are associated with costly treatments and a high degree of patient impairment. However, the pathogenesis of colitis remains poorly understood.

Tumor necrosis factor-alpha (TNF-α) is a potent pro-inflammatory cytokine and increased TNF-α production is found in serum, stools, and bowel mucosa in both IBD patients and IBD models (2). Anti-TNF therapy has been confirmed to alleviate symptoms, heal mucosal ulcers, spare corticosteroid treatment, and decrease hospitalization costs. TNF-α leads to the activation of nuclear factor kappa B (NF-κB), which can transmigrate into the nucleus, and it binds to DNA response elements in gene promoter regions to control transcription of genes, such as inducible NO synthase (iNOS), cyclo-oxygenase-2 (COX2), and myosin light chain kinase (MLCK) (3, 4). Both iNOS and COX2 are pro-inflammatory mediators which play crucial roles in inflammatory responses (3). The expression and activity of MLCK is increased in human IBD and associated with histological evidence of disease activity (5). MLCK-induced phosphorylation of perijunctional actomyosin mediates tight junction loss, which triggers the initiation and development of IBD (6). TNF-α exerts its biological function by binding to two kinds of TNF-α receptors (TNFRs), including TNFR1 and TNFR2. Epithelial TNFR1 and TNFR2 are relatively under-examined, but they have been implicated in epithelial apoptosis, proliferation, migration, and tight junction regulation (7–9).

In general, bromelain is a complex natural mixture of proteolytic enzymes which is derived from pineapple plants (10). Bromelain, a phytotherapeutic drug with the characteristics of efficacy, safety, and lack of undesired side effects after oral administration, has been well accepted in patients (11). Bromelain exerts multiple pharmacological effects, such as preventing edema formation and reducing existing edema, promoting the absorption of antibiotic drugs, affecting blood coagulation and fibrinolysis, as well as anticancer and anti-inflammatory effects (12–14). Oral administration of bromelain relieved IBD symptoms (11, 15, 16); however, the effect of bromelain on intestinal inflammation induced by chemical damage and its underlying mechanisms are still not fully understood.

The present study aimed to examine the effects of purified fruit bromelain (PFB) on TNFRs in a rat colitis model and to determine the role of TNFRs in bromelain-induced alleviation of colitis. The bromelain used in this study is PFB (EC 3.4.22.33, 17 kDa) (17). A rat colitis model was established by intracolonic administration of 2,4,6-trinitrobenzene sulfonic acid (TNBS), which is a classic model for studying IBD (18). The effects of PFB on colitis were also evaluated *in vitro* using the rat intestinal epithelial cell line IEC-6 and human colon epithelial cell line, Caco-2.

## Materials and Methods

### Animals

Fifty-four Sprague-Dawley male rats (5–7 weeks old, weighing between 200 and 220 g) were purchased from the experimental animal center at Dalian Medical University [Certificate of Conformity: No. SYXK (Liao) 2013-0006]. The experimental protocol was approved by the Animal Care and Ethics Committee of Dalian Medical University on June 8, 2012. The animal protocol was designed to reduce pain and discomfort in the animals. The rats were acclimated to laboratory conditions (23°C, 12/12 h light/dark, 50% humidity, *ad libitum* access to food and water) for 2 weeks prior to the experiments. Rats were housed one per cage and they were deprived of food for 12 h before the experiments. All rats were euthanized by barbiturate overdose (intravenous injection, 150 mg/kg pentobarbital sodium) for intestinal tissue collection.

### Reagents

PFB (EC 3.4.22.33) was purified by us from crude proteins of pineapple by high-speed counter-current chromatography (17). Briefly, Matured Pineapple fruits were purchased from a local store (Dalian, China) and authenticated by Dr. Yunpeng Diao (Dalian Medical University, Dalian, China). Pineapple fruit was used to extract the juice, after the juice refined by centrifugation (10,000 × *g*, 30 min, 4°C), finely powdered ammonium sulfate was added into the juice gradually to obtain 50% saturation with continuous stirring for 1 h. Overnight aging at 4°C, some precipitate was separated out and recovered by centrifugation at 10,000 × *g* for 30 min at 4°C. Finally, 7.6 g dry protein was obtained, which was used for subsequently isolation. HSCCC coupled with a reverse micelle solvent system was successfully applied to separate fruit bromelain from fruit of pineapple, and the protein content of separated fraction was reached to 99%, the electrophoresis of obtained fraction purity was 100%, and the activity recovery was 95.5%. Stem bromelain complex (E.C. 3.4.22.32) and sulfasalazine (SASP) were purchased from Tianjin Kingyork Group Co. Ltd. (Tianjin, China). Antibodies to TNFR1 (ab90463), TNFR2 (ab109322), NF-κB (ab16502), MLCK (ab76092), occludin (ab167161), Bcl-2 (ab59348), and Bax (ab53154) were purchased from Abcam Ltd. (Hong Kong, China). Chemicals were obtained from Sigma-Aldrich (St. Louis, MO, USA), unless otherwise indicated.

### Cell Culture

Rat intestinal IEC-6 epithelial cells and human Caco-2 cells were obtained from the cell bank of the Shanghai Institute (Shanghai, China). The cells used in this study were evaluated before the experiments, and no significant interspecies variations in TNFR signaling were observed, which may have affected the results. Cells were maintained at 37°C in a 5% CO_2_ environment. The culture medium consisted of DMEM with 4.5 mg/mL glucose, 50 U/mL penicillin, 50 U/mL streptomycin, 4 mM glutamine, 25 mM HEPES, and 10% fetal bovine serum. Both fetal bovine serum and DMEM were purchased from Invitrogen (Waltham, MA, USA).

### Experimental Design

Twenty-four of the 54 rats were used to test the toxicity of bromelain *in vivo*. The rats were divided randomly into four groups (*n* = 6) including normal control rats, 2.5, 20, and 160 mg/kg PFB-treated groups. The remaining 30 rats were divided randomly into five groups (*n* = 6). The rats were treated as follows: group I, sham-operated control with intracolonic administration of saline; group II, colitis group; group III, SASP (100 mg/kg body weight, intragastric, dissolved in saline); group III, low-dose PFB (10 mg/kg body weight, intragastric, dissolved in saline); group IV, high-dose PFB (80 mg/kg body weight, intragastric, dissolved in saline), 1 day after colitis induction. SASP and PFB were administered by gavage once daily for 14 consecutive days. The rat colitis model was induced as described previously (19). Briefly, rats were fasted for 24 h with free access to drinking water. A catheter was inserted through the anus to approximately the level of the splenic flexure (8 cm proximal to the anal verge) under urethane anesthesia. The colon was then infused with 1 mL of TNBS dissolved in ethanol (50% v/v) at a dose of 125 mg/kg. The rats were allowed to eat and drink *ad libitum* from 1 h after the operation. Distal colon samples from full-thickness intestinal walls were harvested for biochemical studies.

### Assessment of Inflammation

Animal body weight and total food intake for each group were measured daily. Macroscopic colon damage was scored on a scale of 0–10 (20). Colon preparations were stained with hematoxylin and eosin (HE), and the results were evaluated according to previously defined morphological criteria (21–23). Levels of myeloperoxidase (MPO) and pro-inflammatory cytokines were examined using enzyme-linked immunosorbent assay (ELISA) kits (R&D Systems, Minneapolis, MN, USA), according to the manufacturer’s instructions.

### Intestinal Barrier Function Analysis

Intestinal epithelial barrier function was measured *in vivo* according to a previous study (19). Briefly, rats received gavage administration of 150 µL (80 mg/mL) fluorescein isothiocyanate-4 kDa dextran (FD-4) (Sigma-Aldrich, St. Louis, MO, USA), before which rats were fasted but free to water for 3 h. One and three hours later, serum was harvested and measured using a Synergy HT plate reader (BioTek, Winooski, VT, USA). Intestinal barrier function *in vitro* was represented by transepithelial electrical resistance (TER) using an epithelial voltohmmeter. Caco-2 cells (4 × 10^5^) were seeded in the upper chamber of a transwell filter. A barrier dysfunction cellular model was established in Caco-2 monolayers exposed to lipopolysaccharide (LPS).

### Western Blot Analysis

Colon sections were isolated from rats in each group and immediately stored in liquid nitrogen. Total protein was isolated from epithelial layer of the colon section using a Total Protein Extraction Kit (KeyGen Biotech, Nanjing, China). Blots were transferred to nitrocellulose filters and probed with corresponding antibodies at 4°C with gentle shaking overnight. Bands were detected and quantified using a MultiSpectral Imaging system (UVP, Cambridge, UK).

### Cell Transfection

IEC-6 cells were transfected using Lipofectamine 2000 (Invitrogen, Carlsbad, CA, USA) with TNFR1/TNFR2-targeted or control small interfering RNA (siRNA) oligos (Dharmacon, Lafayette, CO, USA), according to the manufacturer’s instructions [Takara Biotechnology (Dalian) Co., Ltd.]. The siRNA sequence for TNFR1/TNFR2 was produced by Genepharma, Ltd. (Shanghai, China). The efficiency of gene silencing was confirmed by western blotting.

### Statistical Analysis

The animal experiments, *in vitro* experiments, and data analyses were conducted according to a single-blind study design. Data were compared among three or more groups using a one-way ANOVA, and between two groups using Student’s *t*-tests. Data were expressed as the mean ± SD. Data were normally distributed and each group showed similar variances. Further evaluations were carried out using Kruskal–Wallis rank sum tests. All experiments were repeated at least six times and a *P* value < 0.05 was considered statistically significant.

## Results

### Dose/Concentration Selection and Toxicity of PFB in Normal Cells and Rats

**Figure 1:**
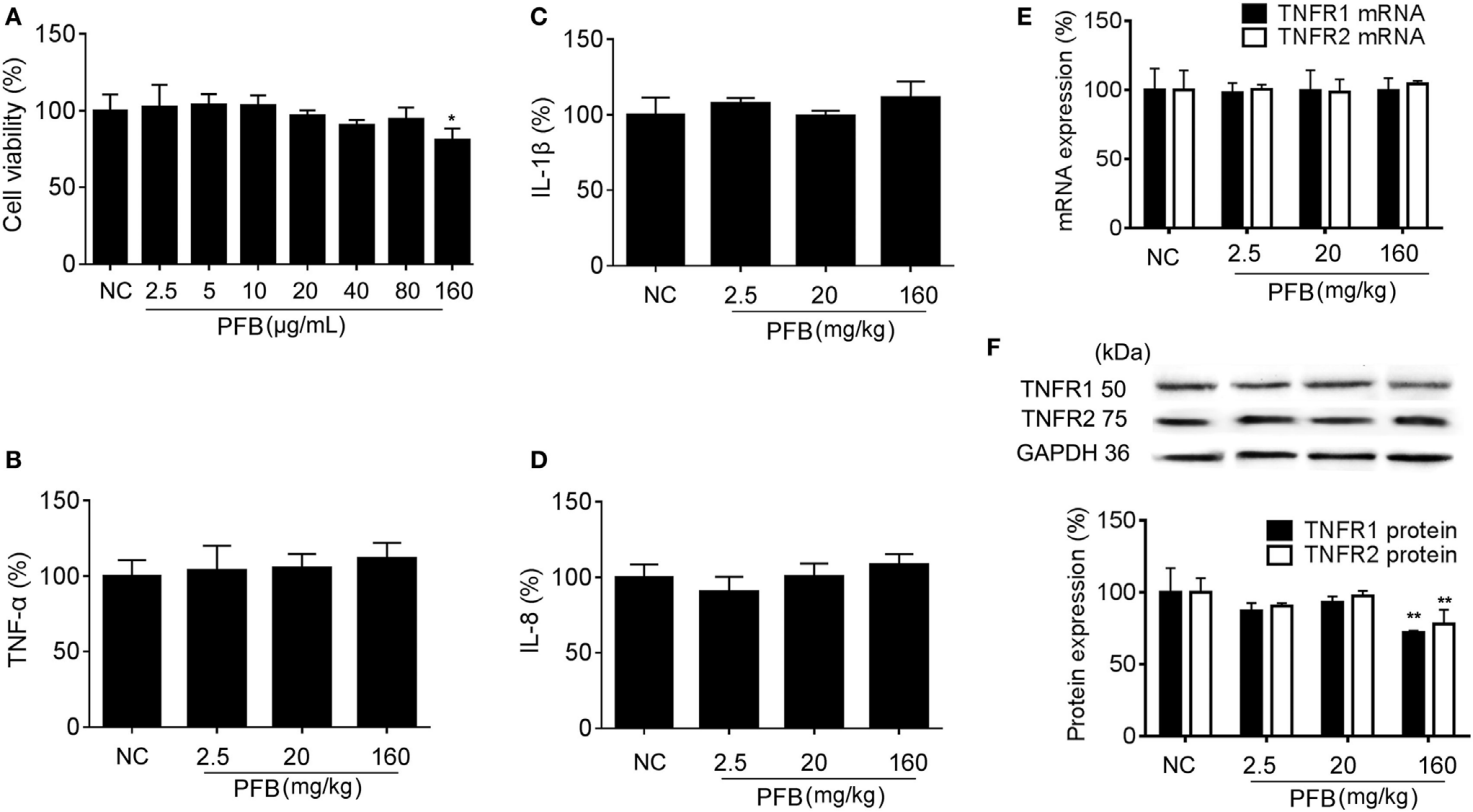
Toxicity of purified fruit bromelain (PFB) in normal cells and rats. **(A)** Cytotoxicity of PFB was studied by MTT assay in the intestinal epithelial cell line IEC-6. PFB was administered to rats by gavage once daily for 14 consecutive days (*n* = 6). Expression levels of the colonic cytokines TNF-α **(B)**, IL-1β **(C)**, and IL-8 **(D)** were examined by enzyme-linked immunosorbent assay, and expression levels of mRNA and protein of TNFR1 and TNFR2 **(E,F)** were examined by quantitative real-time PCR and western blotting, respectively (*n* = 6). Data are expressed as the mean ± SD. Values in the normal control (NC) group are set to 100%, and other values are given relative to the NC group.

The cytotoxicity of PFB in cell lines was examined *in vitro* to determine the effective concentration with no toxicity for subsequent experiments. Exposure to 2.5–160 µg/mL PFB for 36 h had no significant effect on the viability of IEC-6 (Figure 1A). Intragastric administration of PFB (2.5–160 mg/kg) for seven consecutive days had no significant effect on cytokine profiles [TNF-α, interleukin (IL)-1β, and IL-8] (Figures 1B–D) in colon tissue. Furthermore, intragastric administration of PFB had no significant effect on the expression of mRNA and protein of TNFRs (Figures 1E,F). Based on these preliminary experiments and preliminary results reported by other researchers (15, 24), we used <80 mg/kg PFB for *in vivo* and <80 µg/mL PFB for *in vitro* experiments in this study.

### Colitis Rat Morphology and Defecation

Colitis symptoms for rats in TNBS group (TNBS-colitis) are shown in Table 1. Rats in the TNBS-colitis group regained consciousness about 2 h after anesthesia. Symptoms included loose stools, increased stool frequency, outflow of red or dark red liquid from the anus, positive fecal occult blood test, and loss of appetite. On day 3, these symptoms peaked and lasted for about 7 days. Seven days following colitis induction, loose stools were still seen in the TNBS-colitis group. Deaths were recorded throughout the experiment, as outlined in Table 1. New rats were added to maintain six rats per group.

**Table 1:** Colitis rat morphology and defecation.

	Revival time (h)	Symptoms	Symptom duration	Deaths
Sham	1	Loose stools and loss of appetite	First 2 days	0
TNBS-colitis	2	Loss of appetite, loose stools, stool frequency increased, outflow of red or dark red liquid from the anus, positive fecal occult blood test	Symptoms peaked on day 3 and lasted for about 7 days, fecal blood was visible. Loose stools were still seen at 7 days following colitis induction, but fecal occult blood tests were negative	2
SASP	2	Loose stools, increased stool frequency, outflow of red or dark red liquid from the anus, positive fecal occult blood test, and loss of appetite	Symptoms lasted for about 5 days. No fecal blood was visible after 4 days	2
10 mg/kg purified fruit bromelain (PFB)	2	Loose stools, increased stool frequency, outflow of red or dark red liquid from the anus, positive fecal occult blood test, and loss of appetite	Symptoms lasted for about 3 days. No fecal blood was visible after 3 days	1
80 mg/kg PFB	2	Loose stools, increased stool frequency, outflow of red or dark red liquid from the anus, positive fecal occult blood test, and loss of appetite	Symptoms lasted for about 4 days. No fecal blood was visible after 3 days	1

*TNBS, 2,4,6-trinitrobenzene sulfonic acid; SASP, salicylazosulfapyridine*.

### PFB Ameliorated Colitis Symptoms

**Figure 2:**
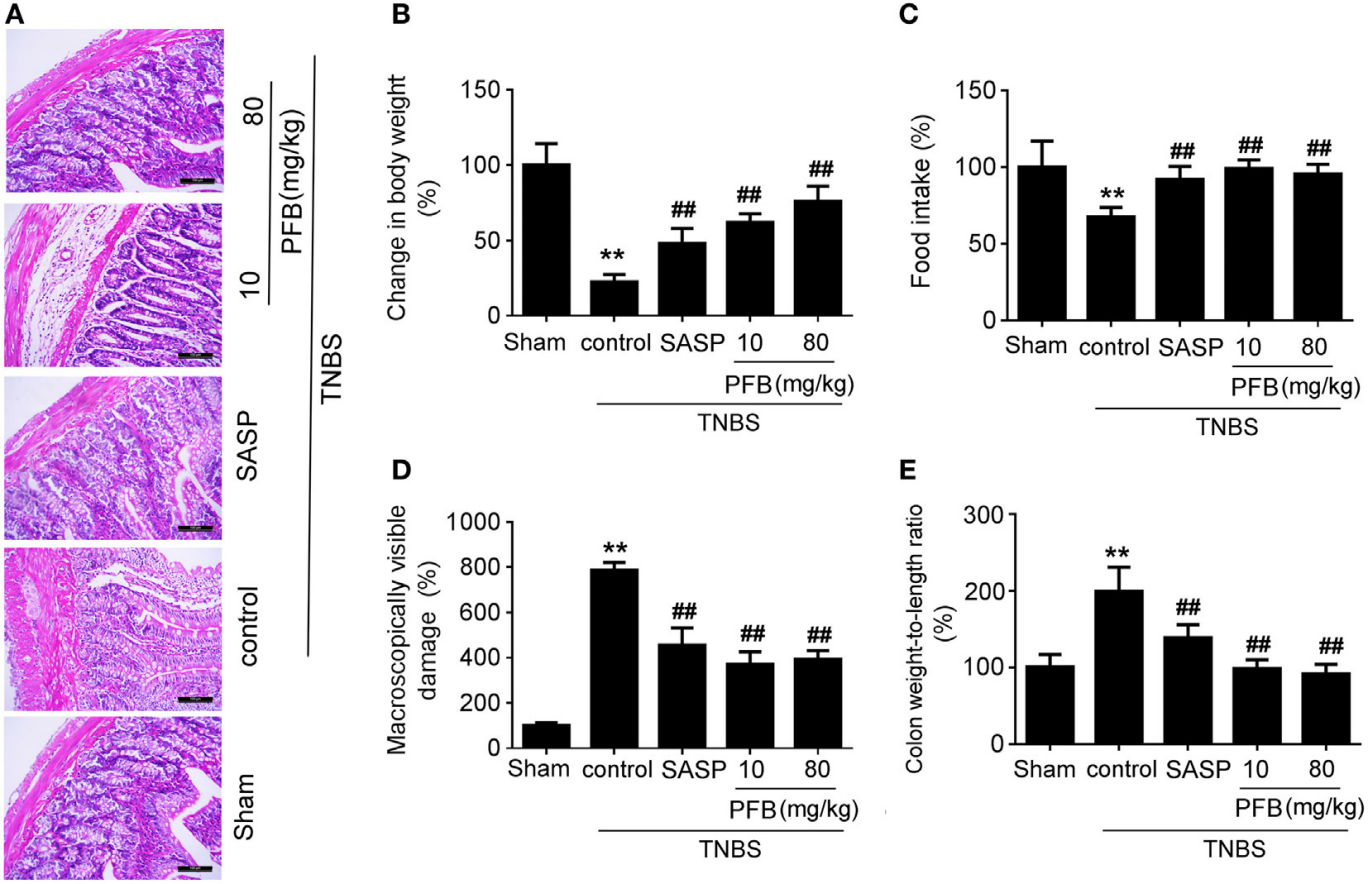
Purified fruit bromelain (PFB) alleviated colitis symptoms. Colitis symptoms were examined after 14 days of PFB therapy. **(A)** Hematoxylin and eosin -staining of rat colonic tissue (20×, scale bar is 100 µm). Effects of PFB on **(B)** body weight, **(C)** food intake, **(D)** macroscopically visible damage, and **(E)** colon weight-to-length ratio in colitis rats. Data in the sham group are set to a relative value of 100% and expressed as the mean ± SD. Other data are the relative values compared with sham: **P < 0.01 compared with sham group (*n* = 6 rats); ^##^*P* < 0.01 compared with TNBS control group (*n* = 6 rats). SASP, sulfasalazine.

**Figure 3:**
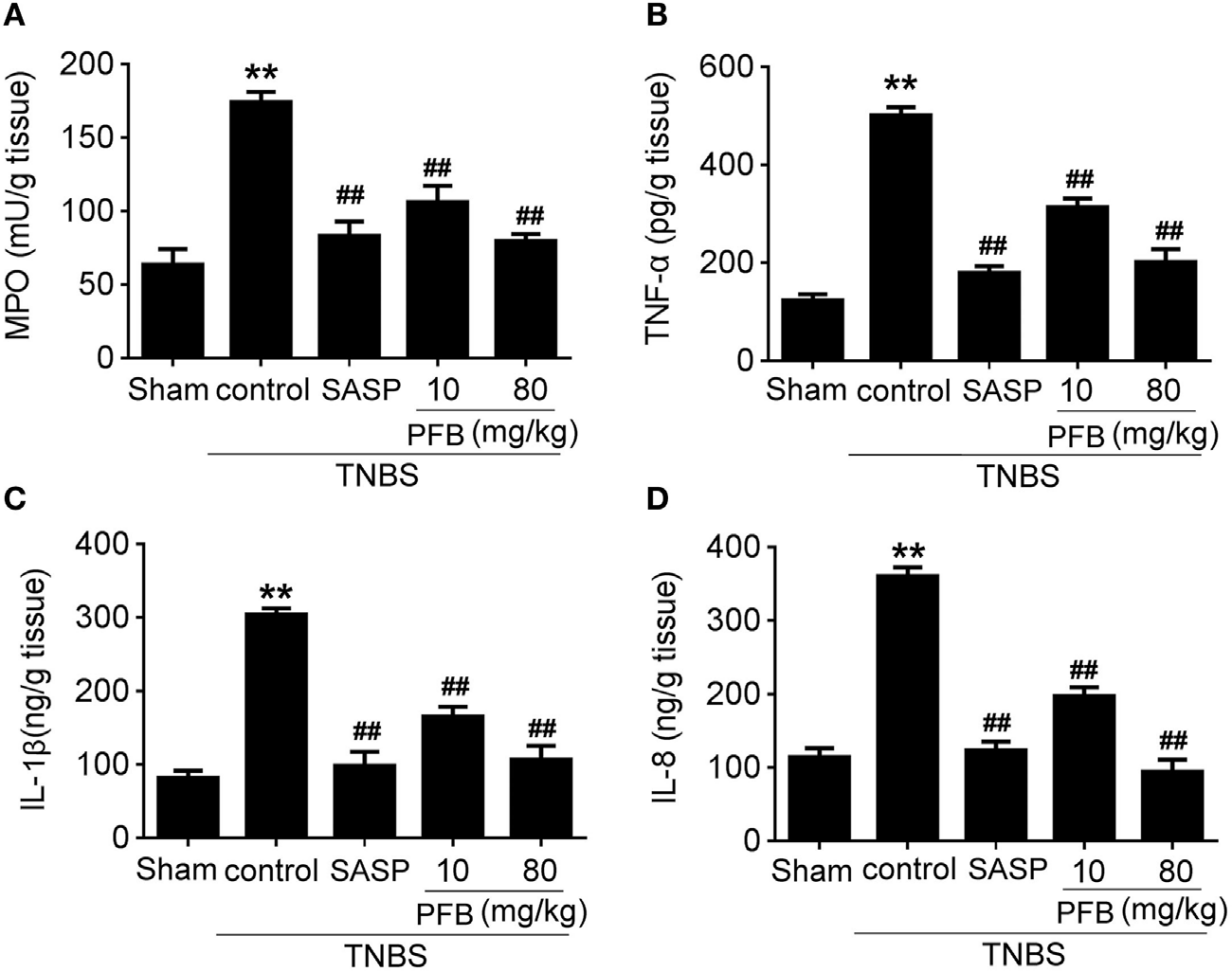
Purified fruit bromelain (PFB) attenuated neutrophil infiltration and cytokine profiles. Effects of PFB on **(A)** myeloperoxidase (MPO), **(B)** TNF-α, **(C)** IL-1β, and **(D)** IL-8 in rat colon tissue determined by enzyme-linked immunosorbent assay. Data are expressed as the mean ± SD; **P < 0.01 compared with sham group; ^##^*P* < 0.01 compared with TNBS control group (*n* = 6 rats). SASP, sulfasalazine.

Successful establishment of colitis was confirmed with biochemical and macroscopic analysis. TNBS challenge provoked apparent colonic mucosal injuries, including serious hyperemia, edema, and ulcers, some of which were covered with necrosis on the surface of colonic mucosa. HE-staining revealed remarkable inflammatory cell infiltration even in muscle layers, irregular arrangement of glands, crypt abscesses, and thickened submucosal edema (Figure 2A). In the colitis group, rats had significantly lower body weight (Figure 2B) and lower food intake (Figure 2C) compared with the sham group, as well as more visible macroscopic damage (Figure 2D) and a higher colon weight-to-length ratio (Figure 2E). Colitis rats also had higher MPO activity (indicating neutrophil infiltration into the damaged tissue), higher pro-inflammatory cytokine levels, including TNF-α, IL-1β, and IL-8. PFB (10 and 80 mg/kg) and SASP reversed the pathological changes in the colitis model after 7 and 14 days of drug treatment, suggesting that PFB significantly ameliorated the colitis symptoms (Figures 2 and 3).

### PFB Restores Intestinal Barrier Function

**Figure 4:**
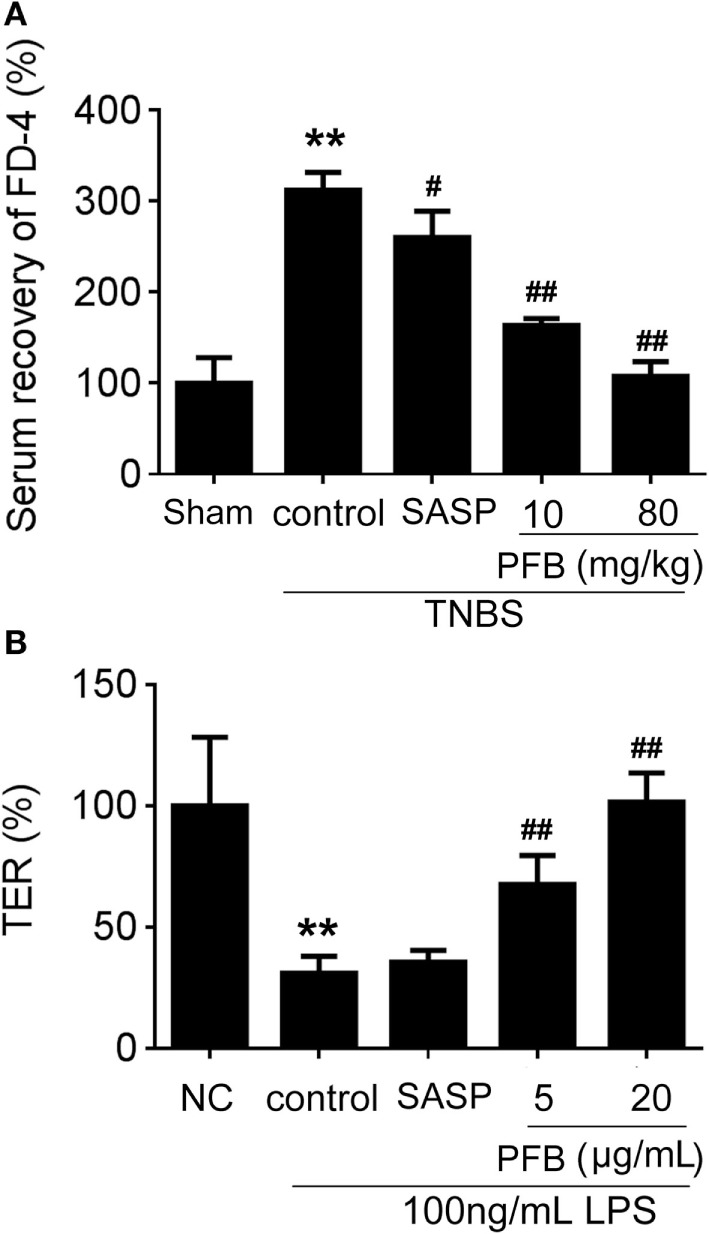
Purified fruit bromelain (PFB) reversed the increased intestinal permeability. After 14 days of PFB therapy, the intestinal permeability in each group was examined through testing serum recovery of FD-4 *in vivo* **(A)** and transepithelial electrical resistance (TER) in Caco-2 cells **(B)** (*n* = 6). Cells were incubated with 100 ng/mL lipopolysaccharide (LPS) for 24 h in the presence or absence of PFB. Data in the sham group or normal control (NC) cell group are set to a relative value of 100% and expressed as the mean ± SD. Other data are the relative values compared with sham or NC: **P < 0.01 compared with sham or NC group; ^##^*P* < 0.01 compared with TNBS control or LPS control group.

*In vivo*, intestinal barrier dysfunction leads to increase in serum recovery of FD-4. In single layer of Caco-2 cells, LPS leads to intestinal barrier dysfunction and significant TER reduction. In this study, the serum recovery of FD-4 was significantly increased in the colitis control group compared with the sham group. Gavage administration of PFB significantly reduced the serum recovery of FD-4. In *in vitro studies*, LPS induced decrease of TER, which was also significantly reversed by PFB (Figure 4). These results suggest that intestinal epithelial barrier dysfunction is recovered by PFB treatment.

### Potential Mechanisms Underlying PFB-Induced Therapeutic Effects on Colitis

**Figure 5:**
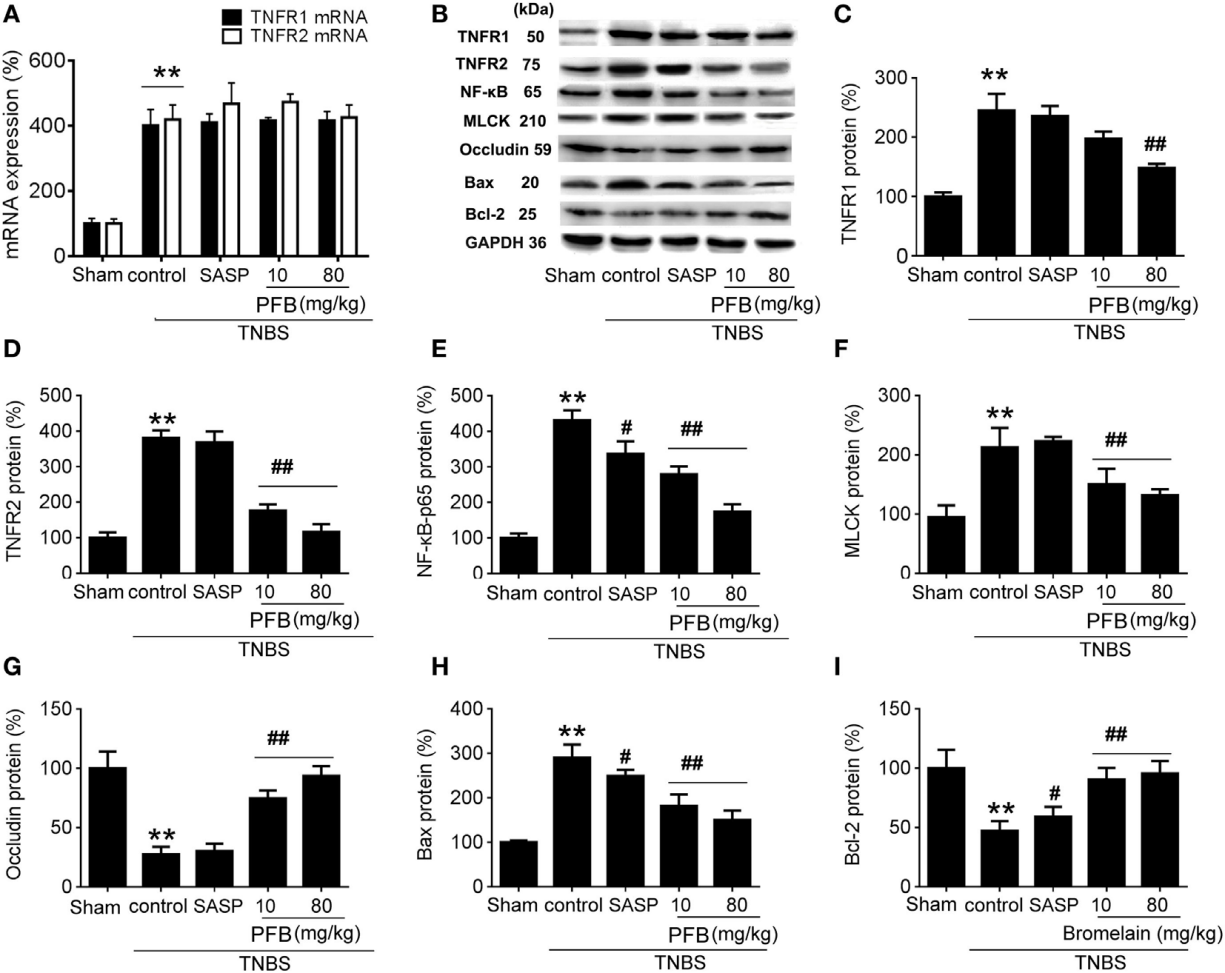
Potential mechanisms underlying purified fruit bromelain (PFB) induced therapeutic effects on colitis. Colonic segments were isolated after 14 days of PFB treatment. Quantitative real-time PCR analysis of TNF-α receptor (TNFR) mRNA **(A)**; Western blotting analysis of TNFR protein **(B–D)**, inflammation marker NF-κB **(B,E)**, tight junction barrier related protein myosin light chain kinase (MLCK) **(B,F)** and **(B,G)**, apoptosis-related proteins Bax **(B,H)** and Bcl-2 **(B,I)**. Data are expressed as the mean ± SD. Values in the sham group are set to 100% and other values are given relative to those in the sham group: **P < 0.01 compared with sham group; ^#^*P* < 0.05 and ^##^*P* < 0.01 compared with TNBS control group (*n* = 6 rats).

In the TNBS group, expression levels of TNFR1, TNFR2, and NF-κB were significantly increased compared with the sham group, and all of these changes were reversed by treatment with PFB (Figure 5). The apoptosis-related protein Bax was significantly increased and Bcl-2 was significantly decreased in colitis rats, and these changes were also reversed by PFB. The epithelial tight junction dysfunction-related protein MLCK was significantly increased and the tight junction protein occludin was significantly decreased, and these changes were also reversed by PFB treatment. However, the increased expression level of TNFR mRNA was not significantly affected by PFB.

**Figure 6:**
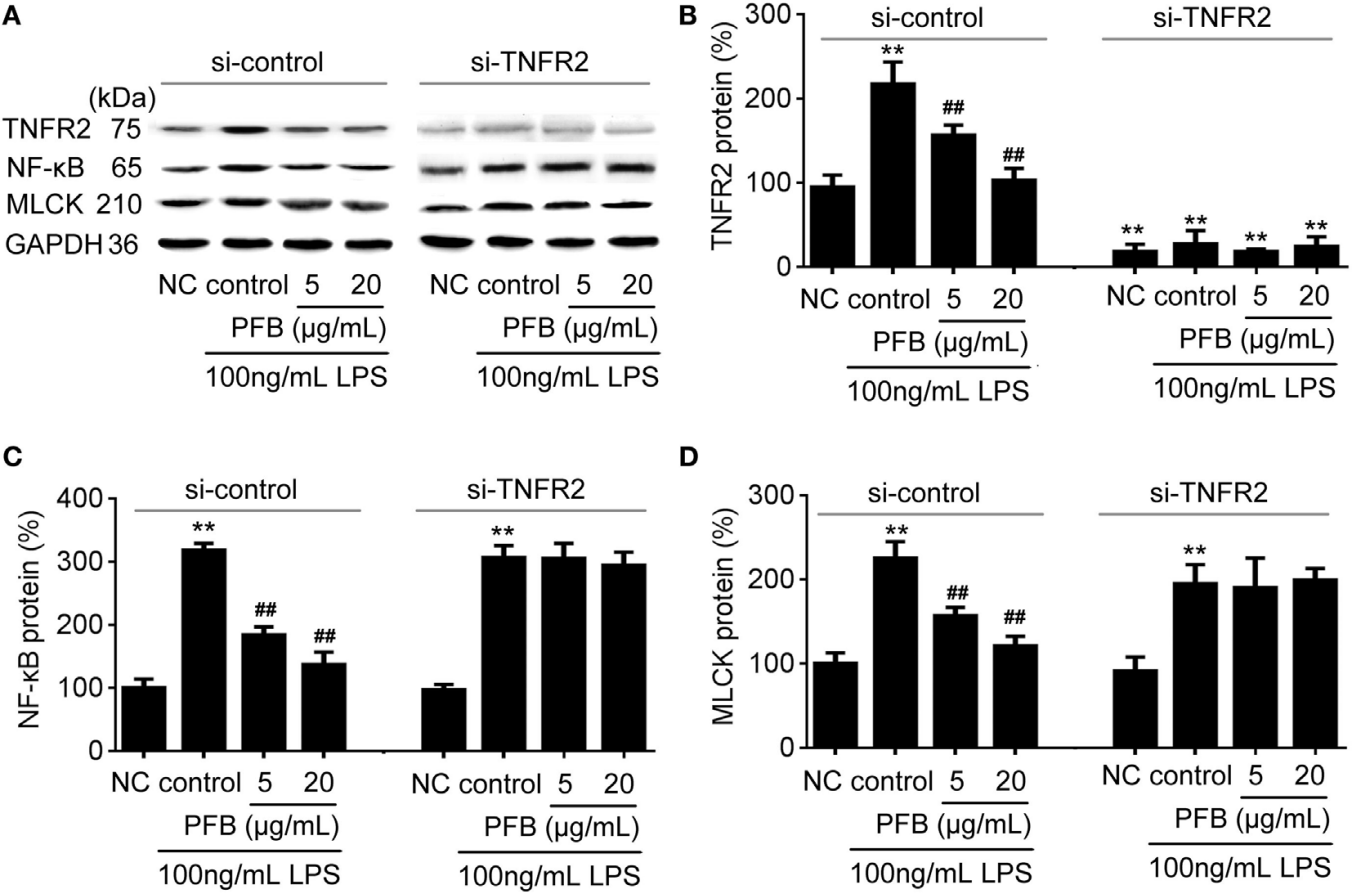
Downregulation of TNFR2 in purified fruit bromelain (PFB)-induced inhibition of inflammation and epithelial barrier dysfunction. Effects of PFB on expression of TNFR2, NF-κB and MLCK in the presence of siRNA targeting TNFR2. **(A)** Representative Western blotting image for effects of PFB on expression of TNFR2, NF-κB and MLCK; statistical analysis of PFB on the expression of TNFR2 **(B)**, NF-κB **(C)**, and MLCK **(D)**. Representative Western blotting image for effects of PFB on NF-κB and MLCK. Data are expressed as the mean± SD. Values are given relative to normal control (NC) in si-control (100%) and other values are given relative to the NC group: **P < 0.01 compared with NC; ^##^*P* < 0.01 compared with corresponding LPS control.

**Figure 7:**
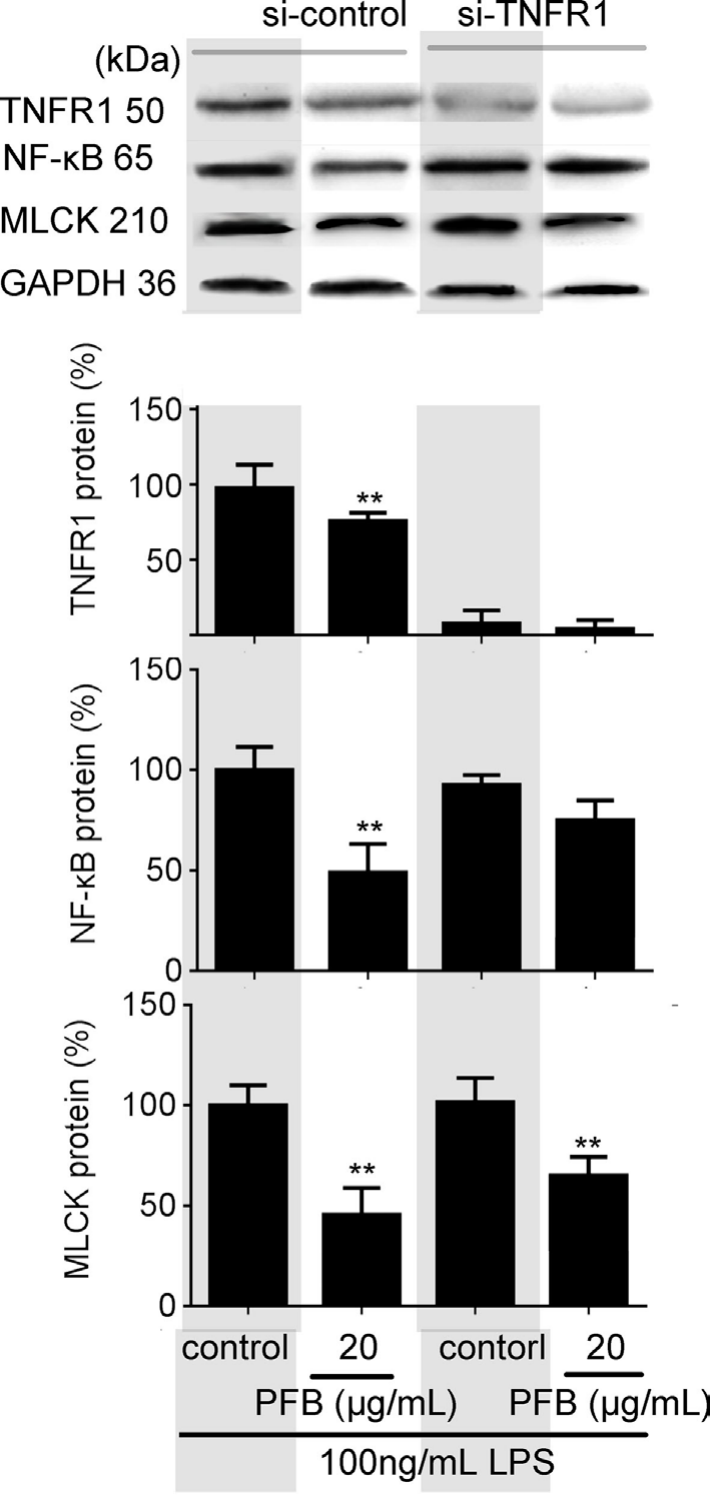
Downregulation of TNFR1 in purified fruit bromelain (PFB)-induced inhibition of inflammation and epithelial barrier dysfunction. Effects of PFB on expression of NF-κB and myosin light chain kinase (MLCK) in the presence of small interfering RNA targeting TNFR1. Data are expressed as the mean ± SD. Values are given relative to LPS-treated group (control, 100%) and other values are given relative to the control: **P < 0.01 compared with LPS control.

To confirm the role of PFB-induced TNFR inhibition in the treatment of colitis, we examined this effect in IEC-6 cells with or without siRNA targeting TNFR2. In normal cells, LPS induced significant increases in NF-κB and MLCK, which reflects the induction of inflammation and epithelial barrier dysfunction. These increases were significantly inhibited by both 10 and 80 mg/kg PFB (Figure 6). The PFB-induced inhibition of NF-κB and MLCK could be significantly abrogated by inhibition of TNFR2 by RNA interference (Figure 6). Moreover, the PFB-induced inhibition of NF-κB but not MLCK could be abrogated by inhibition of TNFR1 by RNA interference (Figure 7), suggesting that PFB alleviated inflammation and epithelial barrier dysfunction in a TNFR2-dependent manner. The results above suggest that TNFR-regulated inflammation, epithelial apoptosis, and tight junction barrier dysfunction in colitis may be blocked by PFB, which in turn may inhibit the release of cytokines, as well as decrease epithelial permeability.

### Comparative Study of PFB with PFB Complex

**Figure 8:**
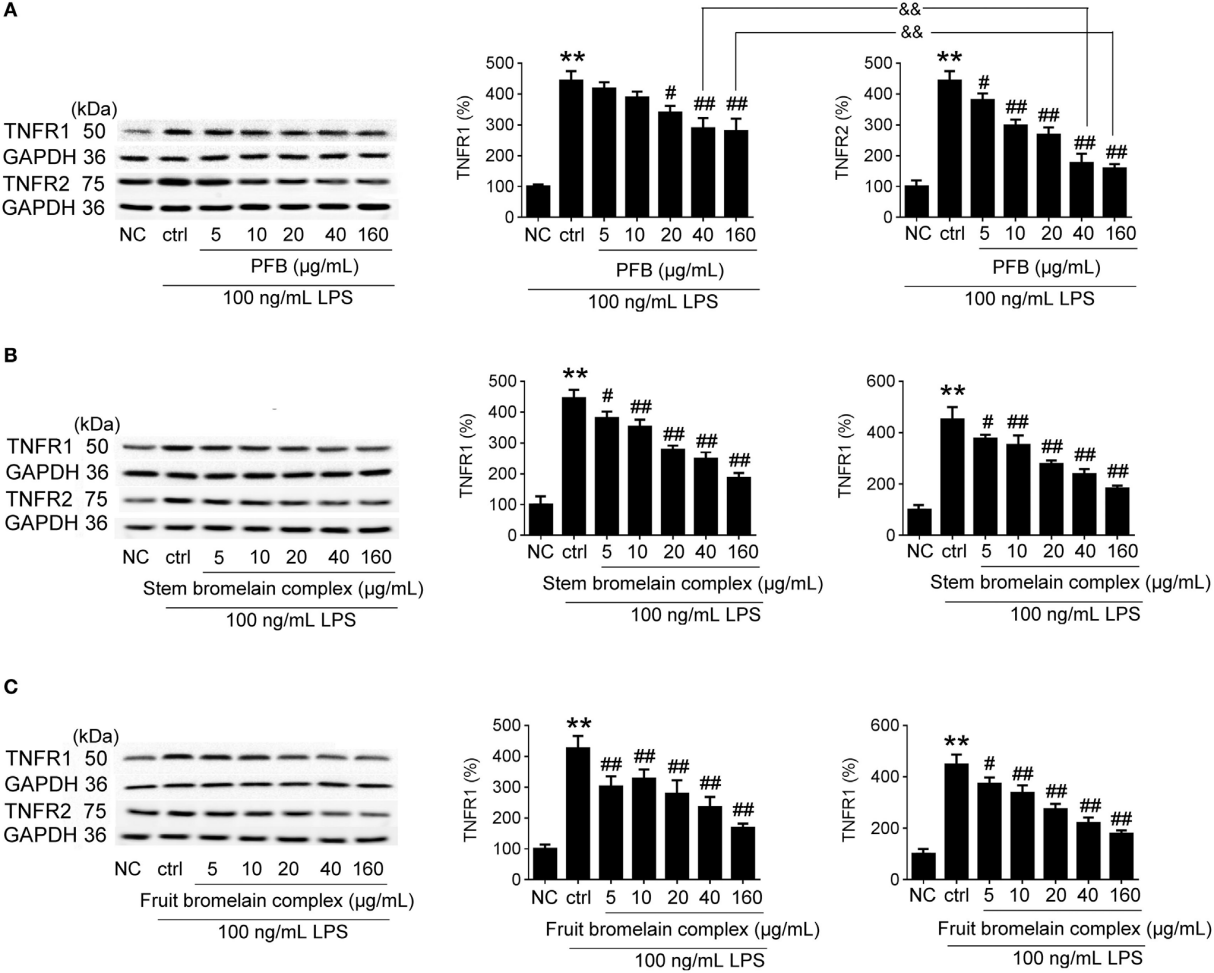
Comparative study of purified fruit bromelain (PFB) with bromelain complex. Western blotting analysis of the effects of purified PFB **(A)**, stem bromelain complex **(B)**, and fruit bromelain complex **(C)** on the protein expression of TNFR1 and TNFR2. Data are expressed as the mean ± SD. Values in normal control (NC) group are set to 100% and other values are given relative to those in the NC group: **P < 0.01 compared with the NC group; ^#^*P* < 0.05 and ^##^*P* < 0.01 compared with LPS control group; ^&&^*P* < 0.01 compared with as indicated (*n* = 6 experiments).

The bromelain used in this study is PFB, but commercial bromelain is a complex natural mixture of proteolytic enzymes derived from pineapple stems (10). The effect of PFB, fruit bromelain complex, and commercial stem bromelain complex on the expression of TNFR1 and TNFR2 in IEC-6 cells stimulated by LPS was studied. Compared with fruit bromelain and stem bromelain complexes, the inhibition of TNFR2 induced by PFB was stronger than the inhibition of TNFR1. These results indicate that PFB showed a stronger selective inhibitory effect on TNFR2 than TNFR1 (Figure 8).

## Discussion

In this study, the expression level of TNFRs including TNFR1 and TNFR2 were significantly increased in a rat colitis model, and PFB-induced reduction of TNFR1 and TNFR2 ameliorated colitis symptoms. Accompanied with an increased expression of TNFR, visible macroscopic damage, mucosal inflammation, and tight junction barrier dysfunction in the colitis model were also significantly increased. PFB reversed the pathological changes in the colitis model.

Gavage administration of PFB significantly decreased colitis symptoms which were indicated by HE-staining, macroscopic damage scores, inflammatory response, as well as recovery of intestinal barrier function. In this study, the epithelial TNFR1 and TNFR2 were mainly studied in a colitis model. Both expression changes of TNFR1 and TNFR2 are involved in the inflammatory response in IBD; however, TNFR2 but not TNFR1 is involved in regulation of tight junction barrier function (25). TNFR1 is involved in the activation of apoptosis through several signaling pathways including MAP kinases and NF-κB activation. The activation of apoptosis process is not regulated by TNFR2 (7). Taken together, TNFR1 and TNFR2 may play distinct roles in IBD, which is also confirmed by our study. Although TNFR1 and TNFR2 play distinct roles in IBD, PFB induced reduction of both TNFR1 and TNFR2 resulting in alleviation of colitis. However, further studies are needed to distinguish the exact role of TNFR1 and TNFR2 in IBD.

Intestinal epithelial barrier dysfunction leads to increased intestinal permeability and finally aggravates colitis (26). High expression level of MLCK, induced by TNFR2 activation, plays an important role in the increase of intestinal permeability (27, 28). In this study, the intestinal barrier dysfunction was significantly alleviated by PFB. TNFR2 expression was significantly reduced by PFB, leading to the reduction of MLCK expression. In the SASP-treated group, the high epithelial permeability was not significantly affected by SASP for 14 days after colitis induction.

Bromelain is one kind of protease whose role is thought to involve degradative action of targets at cell surfaces (29). In this study, increased expression of TNFRs in a colitis model was significantly reversed by PFB treatment. The anti-inflammatory effect of bromelain appears to be related to protease activity. However, other effects such as inhibition of cell growth and metastasis are associated with other nonproteolytic components contained in bromelain (24).

Consideration of two aspects of selectivity helps to clarify the therapeutic effect of PFB in colitis. First is the selective inhibition by PFB of TNFRs and other cell membrane receptors which is related to IBD development, such as toll-like receptors; the other is selective inhibition of the different TNFR isoforms, such as TNFR1 and TNFR2. The selective inhibition of TNFR1 and TNFR2 by PFB was compared with commercial bromelain, which is a mixture of cysteine proteases obtained from both pineapple stems and fruits. Results suggested a stronger selective inhibition of TNFR2 than TNFR1, which confirmed the protective effects of PFB on intestinal barrier function. Thus, the determination of the proteolytic activity alone may not be sufficient to completely characterize the pharmacological properties of bromelain. Results also showed PFB in low concentration indicate a stronger inhibition of TNFR1 expression than TNFR2. We speculated that PFB has a stronger drug potency of inhibition on TNFR1 than TNFR2 in low concentration. Another explanation for this is might be due to the presence of trace level of other proteolytic enzyme; however, it needs further study to examine what kinds of proteolytic enzyme has been involved.

In general, the bioactive constituents of natural sources are more promising candidates for new drug discoveries than specific agonists (30, 31). Purified bromelain is easily obtainable from natural sources because our previous study showed that the content of purified bromelain in pine apple fruit is about 0.15% (g/g). These data showed that purified bromelain is easily obtainable from natural sources and pineapple fruit maybe beneficial for the treatment and prevention of intestinal inflammation. However, the factors including drug stability and controllability in pineapple fruit are not involved. The present study was a preliminary study, with some experimental limitations. The potential toxicological effects of higher concentrations of PFB need to be determined in future studies. The results of the present study provide some novel insights into the mechanisms and potential therapeutic use of the natural product PFB in IBD.

## Ethics Statement

Fifty-four Sprague-Dawley male rats (5–7 weeks old, weighing 200–220 g) were bought from the experimental animal center, Dalian Medical University [Certificate of Conformity: No. SYXK (Liao) 2013-0006]. The experimental protocol was approved by Animal Care and Ethics Committee of Dalian Medical University at June 8, 2012. The animal protocol was designed to reduce pain and discomfort to the animals. The rats were acclimatized to laboratory conditions (23°C, 12/12 h light/dark, 50% humidity, *ad libitum* access to food and water) for 2 weeks prior to the experiments. Rats were housed one per cage and were deprived of food for 12 h before the experiments. All rats were euthanized by barbiturate overdose (intravenous injection, 150 mg/kg pentobarbital sodium) for intestinal tissue collection.

## Author Contributions

DC, YX, JP, YL, and JW designed the research; ZZ, LW, PF, LY, CW, SZ, and JD performed the experiments; ZZ, LW, and PF analyzed the data; DC and YX wrote the manuscript. All authors read and approved the final manuscript.

## Conflict of Interest Statement

The authors declare that the research was conducted in the absence of any commercial or financial relationships that could be construed as a potential conflict of interest.
