# Corrigendum: Inhibition of Epithelial TNF-α Receptors by Purified Fruit Bromelain Ameliorates Intestinal Inflammation and Barrier Dysfunction in Colitis

**DOI:** 10.3389/fimmu.2017.01769

**Published:** 2017-12-13

**Authors:** Zijuan Zhou, Liang Wang, Panpan Feng, Lianhong Yin, Chen Wang, Shengxu Zhi, Jianyi Dong, Jingyu Wang, Yuan Lin, Dapeng Chen, Yongjian Xiong, Jinyong Peng

**Affiliations:** ^1^Laboratory Animal Center, Dalian Medical University, Dalian, China; ^2^College of Pharmacy, Dalian Medical University, Dalian, China; ^3^Dalian Medical University, Dalian, China; ^4^Central Laboratory, the First Affiliated Hospital, Dalian Medical University, Dalian, China; ^5^College of Integrative Medicine, Dalian Medical University, Dalian, China

**Keywords:** bromelain, purification, inflammation, cytokines, myosin light chain kinase, TNF-α receptor, inflammatory bowel disease, tight junction

In the original article, there was an error in a heading in the Results section: **PFB Increases Intestinal Permeability**.

A correction has been made to the name of sub-section 4 in Results, paragraph 4: **PFB Restores Intestinal Barrier Function**.

The authors apologize for this error and state that this does not change the scientific conclusions of the article in any way.

The original article was updated.

## Conflict of Interest Statement

The authors declare that the research was conducted in the absence of any commercial or financial relationships that could be construed as a potential conflict of interest.

